# The Neural Systems of Forgiveness: An Evolutionary Psychological Perspective

**DOI:** 10.3389/fpsyg.2017.00737

**Published:** 2017-05-10

**Authors:** Joseph Billingsley, Elizabeth A. R. Losin

**Affiliations:** ^1^Department of Psychology, University of Miami, Coral GablesFL, USA; ^2^Social and Cultural Neuroscience Laboratory, Department of Psychology, University of Miami, Coral GablesFL, USA

**Keywords:** evolutionary psychology, forgiveness, relationship value, exploitation risk, theory of mind

## Abstract

Evolution-minded researchers posit that the suite of human cognitive adaptations may include forgiveness systems. According to these researchers, forgiveness systems regulate interpersonal motivation toward a transgressor in the wake of harm by weighing multiple factors that influence both the potential gains of future interaction with the transgressor and the likelihood of future harm. Although behavioral research generally supports this evolutionary model of forgiveness, the model’s claims have not been examined with available neuroscience specifically in mind, nor has recent neuroscientific research on forgiveness generally considered the evolutionary literature. The current review aims to help bridge this gap by using evolutionary psychology and cognitive neuroscience to mutually inform and interrogate one another. We briefly summarize the evolutionary research on forgiveness, then review recent neuroscientific findings on forgiveness in light of the evolutionary model. We emphasize neuroscientific research that links desire for vengeance to reward-based areas of the brain, that singles out prefrontal areas likely associated with inhibition of vengeful feelings, and that correlates the activity of a theory-of-mind network with assessments of the intentions and blameworthiness of those who commit harm. In addition, we identify gaps in the existing neuroscientific literature, and propose future research directions that might address them, at least in part.

## Introduction

Evolutionary researchers hypothesize that human social decision-making relies upon cognitive adaptations designed by natural selection to optimize fitness outcomes in the ancestral environment (Tooby and Cosmides, 2005). Such adaptations may include forgiveness mechanisms, cognitive systems which evolved to address the difficult cost-benefit challenges posed by intricate social interactions (McCullough, 2008; McCullough et al., 2013). According to a model of forgiveness proposed by evolutionary theorists (McCullough, 2008; McCullough et al., 2013), these forgiveness systems regulate individual motivation toward a transgressor by weighing the many factors that influence both the potential gains of future interaction and the likelihood of future harm. Depending on the outcome of these computations, the victim may experience forgiveness. Forgiveness is understood in this model as a shift in interpersonal motivation, marked by reduced retaliatory sentiment, decreased avoidant sentiment, and/or increased goodwill toward the transgressor. This shift in interpersonal motivation has the ultimate purpose of realizing long-term benefits of continued, productive interaction, and may be contingent upon improved treatment by the transgressor (McCullough et al., 2013).

Empirical tests of the evolutionary forgiveness model thus far have frequently been longitudinal in nature (McCullough et al., 2010, 2014), although recent experimental work using both cognitive priming (Burnette et al., 2012) and a behavioral economics framework (Tabak et al., 2012), has generally supported the model. The neuroscientific literature, meanwhile, has begun to identify the neural correlates of social decision-making across an array of contexts, including those involving forgiveness, but rarely engages this evolutionary model of forgiveness directly. Here we briefly summarize the evolutionary research on forgiveness, then review recent neuroscientific findings on forgiveness in light of the evolutionary model—with the aim of using each body of work to interrogate and inform the other. We conclude by outlining directions for future research based on gaps in the existing literature.

## An Evolutionary Approach to Forgiveness

Evolutionary treatments of forgiveness begin with an adaptive problem reasonably assumed to characterize the ancestral human environment: exploitation of individuals by conspecifics (McCullough, 2008; McCullough et al., 2013). Whether in large-scale Western societies or among traditional foragers, the catalog of human exploitation is all too diverse: rape, pillage, extortion, murder, infidelity, kidnap, assault, and now even identity theft dot its pages (Buss and Duntley, 2008). Furthermore, an archeological record littered with evidence of human violence suggests that the use of force to prey upon others was, if anything, more prevalent ancestrally than at present (Pinker, 2011). And humans are certainly not the only species to exploit conspecifics: the animal behavior literature documents a wealth of species—including our close primate relatives—in which individuals frequently gain fitness benefits for themselves by imposing high costs upon others in the social group (Clutton-Brock and Parker, 1995).

Comparative examination of social species marked by conspecific exploitation suggests a common adaptive response to the threat: punishment, defined straightforwardly as retaliatory action taken in response to the costly (fitness-reducing) behavior of another (Clutton-Brock and Parker, 1995). A victim who directs punishment at an exploiter alters the cost/benefit equation for that exploiter; any fitness gains realized by the exploiter must then be discounted, and may even turn gains to losses if punishment is severe enough. Evidence from numerous species, including cowbirds and such primates as hamadrayas baboons, vervet monkeys, bonnet macaques, and chimpanzees, indicates that punishment frequently provides benefits by modifying the future behavior of the transgressor, essentially teaching the transgressor to engage in behaviors more beneficial, or less costly, to the punisher (Clutton-Brock and Parker, 1995; McCullough et al., 2013). Depending on the cognitive capacities of the species, punishment may also have a rippling reputational effect, discouraging exploitation at the hands of third-party observers (McCullough et al., 2013). Against this background, the human motivation for revenge emerges not as a disease, as it is so often conceptualized (McCullough, 2008), but as an adaptation which, at least in the ancestral environment, likely functioned to deter exploitation.

The functional benefits of revenge in an ancestral context seem clear enough, but revenge often entails secondary costs that pose an adaptive problem of their own (McCullough et al., 2013). Not only might revenge set in motion a deadly feedback loop of unending counter-retaliation (Boehm, 1987), but vengeance might also terminate long-standing and potentially productive relationships (McCullough, 2008; Burnette et al., 2012; McCullough et al., 2013). Although retribution may result in better treatment in the future, it may also dissolve the relationship entirely, and with it an entire stream of future net benefits (McCullough, 2008; Burnette et al., 2012; McCullough et al., 2013). An alternative solution to harm is to seek to preserve the relationship and the future benefits it offers, while taking other, non-punitive steps to ensure that similar harms do not recur. In this context, forgiveness emerges in the wake of a transgression as a redirection of motivation away from punitive or avoidant inclinations, toward greater benevolence, with the ultimate aim of securing continued, benefit-producing interaction with the transgressor (McCullough et al., 2013).

On this view, revenge and forgiveness are thus intertwined aspects of cognitive systems designed to navigate the difficult terrain of complex social interaction. According to this evolutionary model, cognitive mechanisms for revenge and forgiveness function to optimize fitness outcomes resulting from engagement in a web of relationships that offer both the promise of cooperative interchange and the peril of malicious exploitation. Researchers developing this model have sought to identify the computational requirements implied by such systems, and have employed the cost/benefit reasoning described above to suggest that cognitive mechanisms well-designed for making decisions about when and whom to forgive should incorporate information of two broad types. First, such systems should assess cues relevant to the *relationship value* of the transgressor, defined as the net fitness benefits expected to result from continued interaction with the transgressor. Second, they should weigh cues indicating *exploitation risk*, defined as the likelihood that the transgressor will impose future costs on the victim (McCullough, 2008; McCullough et al., 2010, 2013, 2014; Burnette et al., 2012; Petersen et al., 2012).

Empirical work on this model has formulated and validated the Relationship Value and Exploitation Risk (RVEX) scale (Burnette et al., 2012), and used it to investigate several hypotheses: (1) that the relationship value of the transgressor is *positively* associated with forgiveness; (2) that the exploitation risk posed by the transgressor is *negatively* associated with forgiveness; and (3) that the interaction of relationship value with exploitation risk will predict forgiveness, such that the greatest levels of forgiveness will be directed toward transgressors who are both high in relationship value and low in exploitation risk. To test these hypotheses, Burnette et al. (2012) obtained two separate measures of forgiveness from a sample of more than 350 undergraduate students currently in a romantic relationship. One measure, the Exit/Neglect/Voice/Loyalty Scale (ENVL; Rusbult, 1993), operationalized forgiveness based on responses to hypothetical acts of betrayal committed by the participant’s romantic partner; a second measure, the Transgression Related Interpersonal Motivations inventory (TRIM; McCullough et al., 1998), operationalized forgiveness as the degree of vengeful, avoidant, or benevolent motivation reported by participants who recalled and reflected upon the most damaging thing that their romantic partner had done to them in the previous 3 months. Burnette et al. (2012) found that exploitation risk and the interaction of exploitation risk with relationship value predicted both the ENVL and TRIM measures of forgiveness in the expected directions, controlling for a number of variables, including trait forgiveness, empathy, relationship commitment, time since transgression, and offense severity. Likewise, relationship value predicted the TRIM measure of forgiveness but not the ENVL measure, controlling for the same variables. Using a separate, online sample, Burnette et al. (2012) randomly assigned more than 400 participants to think either about a high-value or a low-value associate, then primed each participant to focus on exploitive or non-exploitive aspects of the relationship. With this manipulation, and using the TRIM inventory as their sole dependent variable, Burnette et al. (2012) found support for all three key variables—relationship value, exploitation risk, and their interaction—as predictors of forgiveness. Employing a longitudinal design with 337 participants recently harmed by someone close to them, McCullough et al. (2014) operationalized forgiveness as latent change in TRIM scores over a 3-week period. Results confirmed that apologies and other conciliatory gestures made by transgressors were associated with both increased reported relationship value and decreased exploitation risk. Although in this study the interaction of relationship value with exploitation risk did not predict forgiveness, both relationship value and exploitation risk mediated the effect of conciliatory gestures upon forgiveness (McCullough et al., 2014).

Ongoing research seeks to elaborate upon the specific types of information that contribute to relationship value and exploitation risk. Relationship value is linked to such predictors as the status, resources, or sexual value of the transgressor; the nature and magnitude of past benefits provided to the victim; the length of the prior relationship; and the transgressor’s future prospects. Exploitation risk is tied to such factors as the severity of the offense; indicators that the transgressor acted intentionally; sincere expressions of regret including apologies, acts of contrition, and costly compensatory offers; observed or reputed past behavior likely to presage future harm; and other cues that the transgressor might be trusted not to inflict further costs (Petersen et al., 2010; Burnette et al., 2012).

If forgiveness results from evolved cognitive mechanisms for making ancestrally adaptive social decisions, then the computational architecture of these mechanisms must be instantiated in neural tissue. The proponents of the evolutionary model reviewed above deliberately refrain from addressing any neural correlates of the putative systems they describe, but they note that “as imaging technology becomes more powerful and theorizing about the interface of cognitive science and neuroscience becomes more sophisticated, cognitive neuroscientists will increasingly be in a position to shed light on the neural bases of the computational systems we have presented here” (McCullough et al., 2013, p. 48). The cognitive neuroscience of forgiveness assuredly still remains in its infancy. Nonetheless, a small but slowly burgeoning neuroscientific literature has begun to clarify the neural mechanisms underlying forgiveness and revenge. In what follows, we review key findings from this literature in light of the evolutionary model presented above. This evolutionary model is grounded in a tight linkage between revenge and forgiveness, and one that grants temporal priority to punitive sentiment. Accordingly, we begin by examining the neural bases of vengeance.

## The Neural Bases of Vengeance

Numerous imaging studies suggest that the striatum is typically activated in reward-processing. Activation of the striatum, particularly the nucleus accumbens and the caudate nucleus, is closely associated with motivation to engage in behaviors linked to the promise of reward (Schultz, 2000; Cardinal et al., 2002; O’Doherty et al., 2004; Delgado, 2007). Converging evidence from social neuroscience suggests that the striatum plays a role in motivating behavior directed not only toward tangible rewards such as prized food items or addictive drugs (Delgado, 2007) but also toward desired social outcomes (Bhanji and Delgado, 2014), including revenge (Rilling and Sanfey, 2011). Let us be clear here that activity in the striatum and other areas associated with reward-processing does not imply hedonic value *per se* but rather a more general association with reinforcement of certain behaviors over others (Schultz, 2000).

One of the first studies to suggest an association of the striatum with vengeful motivation was conducted by de Quervain et al. (2004), who employed positron emission tomography (PET) to observe the brain activity of subjects engaged in a modified version of a behavioral economics task known as the Trust Game (TG). In this task, the participant (the “investor”) is invited to transfer some or all of an endowment to a second player (the “trustee”). Any transferred funds are multiplied, after which the trustee may (or may not) return half of the new total to the investor. Thus, if the trustee can be relied upon to reciprocate, the investor maximizes returns by transferring all of the endowment, but in doing so runs the risk of being cheated. de Quervain et al. (2004) elaborated upon this basic game structure by allowing investors to punish untrustworthy partners, either by deducting funds from the trustee’s earnings or by assigning symbolic punishment points. PET scans conducted while investors decided whether or not to punish revealed activation of the caudate nucleus, with particular elevation observed in conditions where participants could impose monetary rather than merely symbolic costs on trustees. Moreover, stronger activation of the caudate was associated with greater investment in punishment (de Quervain et al., 2004). In light of studies implicating this region in reward-related processing, researchers suggested that punishment of trust violations may be motivated by an anticipated reward associated with the prospect of inflicting punishment, and that caudate activity may index the magnitude of this anticipated reward (de Quervain et al., 2004; Knutson, 2004).

Using functional magnetic resonance imaging (fMRI) and a different behavioral economics paradigm, Brüne et al. (2013) found evidence corroborating the notion that the striatum is associated with motivation for revenge, although their results implicated more ventral areas of the striatum. To investigate punishment, Brüne et al. (2013) employed an Ultimatum Game (UG) followed by a Dictator Game (DG). The UG features two players, the first of which (the “proposer”) is given an endowment and then makes an offer to the second player (the “responder”) regarding how the money should be divided between them. The responder may accept the offer, in which case each partner receives the proposed allocation, or the responder may reject the offer, in which case both parties receive nothing. Although rational choice theory from economics would predict that proposers offer virtually nothing, and responders accept any non-zero offer, empirical results show that many offers approach a 50/50 split, and rejection rates rise as offer amounts fall (Camerer, 2003). The DG is a bit simpler: the first player (in the role of “dictator”) is endowed with a sum of money and may give some, all, or none of it to the second player (the “recipient”); unlike responders in the UG, recipients have no say in what happens and simply accept what the dictator proposes. Subjects in the Brüne et al. (2013) experiment first took the role of responder in interactions with an array of both consistently fair and consistently unfair human partners, as well as with a computer playing randomly. Subjects then assumed the role of dictator in the DG and were required to allocate money among themselves and the other players whom they have previously faced in the UG. Punishment was defined as low DG allocations made to previously unfair UG Proposers. Brüne et al. (2013) found increased right ventral striatal activation when participants punished unfair partners in the DG, vs. when participants treated fair players equitably. In the UG, moreover, level of activity in the participant’s right ventral striatum positively correlated with the rate at they rejected offers, which in turn correlated with the unfairness of offers (Brüne et al., 2013), consistent with the suggestion that punishment may be associated with reward.

A study conducted by Singer et al. (2006) offered further support for the notion that revenge-based motivation and punishment activate reward-related regions, specifically in the left ventral striatum/nucleus accumbens. Participants in Singer et al.’s (2006) study played an iterated sequential Prisoner’s Dilemma (PD) game with two researcher confederates—one who acted fairly, the other unfairly. Following the PD game, participants’ brain activity was recorded with fMRI while the participant observed the hands of all three players receiving electrical stimulation, either intense (pain condition) or mild (no pain condition). Singer et al. (2006) found elevated activation of the left ventral striatum/nucleus accumbens when men (but not women) observed unfair vs. fair players subjected to pain. The finding is suggestive: Might activation of the striatum in this context be associated with punitive motivations tied to the other player’s earlier unfair behavior? Self-report data collected by Singer et al. (2006) indicate this is likely the case: men’s expressed revenge motivation predicted nucleus accumbens activity when observing unfair players subjected to pain.

Singer et al.’s (2006) finding of a gender difference in revenge-related striatal activation possibly bears on suggestions from the behavioral literature on forgiveness that women may be more forgiving than men, and display less vengeful motivation (Miller et al., 2008), though a meta-analysis by Fehr et al. (2010) failed to support the claim. If the gender difference in vengeful motivation proves robust, Singer et al.’s (2006) research provides a possible proximate explanation: the increased vengeful motivation of men relative to women following unfair treatment is associated with stronger activation of reward-related areas of the brain in response to the prospect of inflicting punishment.

Distinctive neural responses accompanying punishment, however, are not limited to the striatum (Rilling and Sanfey, 2011), suggesting that punitive decisions involve more than processing potential rewards. Indeed, many neuroscientific studies involving punishment have demonstrated activation of the anterior insula (AI), a region associated with representation of bodily states (such as hunger, thirst, and touch; Craig, 2002, 2003, 2009; Critchley, 2009) as well as with multiple emotions, particularly negative emotions such as disgust (Phillips et al., 1997; Calder et al., 2000; Wicker et al., 2003; Vytal and Hamann, 2010) and anger (Lindquist et al., 2012). The association of the AI with negative emotional responses to aversive stimuli may explain observations of elevated AI activity in participants being treated unfairly by others. Sanfey et al. (2003), for instance, observed heightened activation of the bilateral AI [along with the dorsolateral prefrontal cortex (dlPFC) and anterior cingulate cortex] in conjunction with unfair vs. fair UG offers from human partners. Activation in the insula was greater in response to unfair offers from human vs. computer partners, suggesting that the pattern of response was not a function of low offers in general, but more specific to human unfairness. And the bilateral insula in particular appeared to track the degree of unfairness: the lower the offer, the more elevated the insular activity. What’s more, magnitude of insular activity predicted likelihood of rejecting an unfair offer, both between and within subjects. This set of findings suggests that AI activity in this context may index negative emotional responses to unfair treatment (Sanfey et al., 2003). Other researchers have produced similar results. In another fMRI study of participants acting as responders in an UG, Tabibnia et al. (2008) found that anterior insular activity was heightened in response to offers that the participants themselves deemed unfair. Moreover, when participants accepted rather than rejected such unfair offers, the left AI exhibited reduced activity, consistent with the possibility that they may have experienced less aversion to the unfairness. Brüne et al.’s (2013) fMRI study of punishment, described previously, likewise found that bilateral activity in the AI accompanied activation of the ventral striatum when UG responders encountered unfair offers, again providing a possible association of the AI with negative responses to unfairness.

Departing slightly from the methods of behavioral economics, Will et al. (2014) examined the neural correlates of punishment by using a virtual ball-tossing game—Cyberball—to induce feelings of exclusion, ill-will, and anger in participants. Following their Cyberball experience, participants assumed the role of Dictator in a DG, and were forced to choose between various distributions of money to Cyberball players who had either included them in or excluded them from the ball-tossing. fMRI scans conducted during the decision-making interval revealed that punishment—operationalized in this study as selection of outcomes resulting in less-than-equal payouts to excluders—was linked to heightened activation of the AI (along with the pre-supplementary motor area), just as occurred with unfair offers in the UG studies reviewed above (Will et al., 2014).

Given that the insula is not generally implicated in the reward system, how might we interpret its role in punishment? Broader neuroscientific research suggests that the insula serves many functions but appears to be tightly linked to the representation of somatic and visceral states (Craig, 2009; Singer et al., 2009; Chang et al., 2012). Both imaging and lesion studies reveal a role of the insula in interoceptive representation of such somatic states as pain, hunger, and taste, as well as negative emotions such as disgust and anger (Phillips et al., 1997; Calder et al., 2000; Calder et al., 2001; Craig, 2002, 2003, 2009; Wicker et al., 2003; Singer et al., 2009). Research also suggests that insular involvement extends to the social domain (Rilling and Sanfey, 2011), including empathic responses to the pain, tastes, and disgust experienced by others (Wicker et al., 2003; De Vignemont and Singer, 2006; Singer and Lamm, 2009), as well as visceral aversions to morally repugnant actions (Greene, 2009). This array of evidence regarding the role of the insula in representing negative emotion and somatic states, particularly aversion, suggests that in the punishment studies reviewed above the insula serves as a locus of negative affect, delivering an aversive response—perhaps realized at a visceral level—to the unfair, fitness-damaging behavior of others. Co-activation of the AI and the striatal reward circuits following unfair treatment plausibly suggest the notion of a negative reaction associated with the AI and an accompanying desire for retribution associated with the striatum.

## The Role of Inhibitory Networks in Forgiveness

If revenge and punishment are associated with heightened activation of the striatum and the AI, what brain mechanisms accompany forgiveness, the flip-side of vengeance? Although it might be reasonable to expect that motivations to forgive would also be driven by reward centers of the brain, much evidence suggests otherwise; in fact, forgiveness may involve *inhibition* of the AI and striatum, primarily driven by prefrontal cortical regions. Evidence again comes from the work of Brüne et al. (2013), who took fMRI scans of subjects making DG allocations to individuals who had previously treated them either fairly or unfairly in an UG. In this study, high DG allocations to unfair UG proposers indicated more forgiving, less punitive behavior toward a transgressor. As noted previously, Brüne et al. (2013) documented striatal activation in conjunction with participants receiving unfair offers during the UG game, and in conjunction with participants subsequently making *low* DG allocations to previously unfair players. Such findings are consistent with the research reviewed above suggesting a link between retaliatory motivation and reward. No striatal activation, however, was detected when individuals made *high* DG allocations to previously unfair players—suggesting that more forgiving responses to unfairness, at least in this context, may not involve a reinforcement signal akin to that experienced in association with retaliation. Instead, Brüne et al. (2013) found that these high DG allocations to previously unfair individuals were associated with increased activation of the dlPFC, possibly indicating cognitive control of aversive responses to unfairness (Brüne et al., 2013).

A variety of other studies have likewise reported lateral prefrontal activity in conjunction with more benevolent, less punitive responses to unfair treatment. In their fMRI study of reactions to Cyberball-induced social exclusion, Will et al. (2014) observed amplified activity of the dlPFC, ventrolateral prefrontal cortex (vlPFC), and dorsal anterior cingulate cortex (dACC) in subjects who did not punish Cyberball players who had excluded them, compared to subjects who did punish excluders. Increased activation of the dlPFC relative to the AI was also observed by Sanfey et al. (2003) in their fMRI studies of UG players, specifically when players accepted unfair offers. By contrast, when players *rejected* unfair offers, AI activity was heightened relative to that of the dlPFC, leading Sanfey et al. (2003) to suggest that dlPFC activity in this context indexed regulatory control over punitive inclinations. Lateral prefrontal activity in conjunction with the acceptance of unfair UG offers was also observed by Tabibnia et al. (2008), but in their study it was the vlPFC rather than dlPFC that showed increased activation when unfair UG offers were accepted. Elevated activation of the dlPFC in the context of forgiveness was also noted by Ricciardi et al. (2013). Ricciardi et al. (2013) performed fMRI scans of subjects who were first asked to imagine themselves in hurtful social scenarios, and were then encouraged either to forgive the harmer or to dwell on revenge. Contrasts revealed that the forgiveness condition was associated with differential activation of the dlPFC [along with regions implicated in a theory-of-mind network (Premack and Woodruff, 1978), thought to be involved in inferring the mental states, beliefs, and intentions of others]. A wealth of social neuroscientific studies provide evidence linking the lateral prefrontal regions with emotional regulation, impulse control, and other inhibitory activity (Miller and Cohen, 2001; Aron et al., 2004; Ochsner and Gross, 2005; Lieberman, 2007; Greene, 2009; Rilling and Sanfey, 2011). This evidence in conjunction with the work reviewed here suggest that, in the context of forgiveness, the dlPFC and vlPFC, as well as the dACC, may act as part of a network down-regulating negative affective responses of the AI to unfairness and other social harms, while also inhibiting the punitive motivations generated by the striatal reward centers of the brain (Rilling and Sanfey, 2011; Brüne et al., 2013; Ricciardi et al., 2013).

## The Role of Theory of Mind in Forgiveness Mechanisms

Evolutionary perspectives on forgiveness suggest that victims of interpersonal injury calculate the probability of future harm at the hands of the transgressor (“exploitation risk”) and integrate this variable into the decision-making process (Petersen et al., 2010; McCullough et al., 2013). The likelihood of future harm hinges, in turn, on the intentions of the transgressor, both past and present. If victims are to respond to interpersonal harms adaptively, they must assess transgressor intentions accurately (Burnette et al., 2012). The consequences of error in this domain are potentially severe. To attribute malign intent to a long-term cooperative partner who harmed you inadvertently could mean needlessly sacrificing years of productive interchange—forgoing access to resources, crucial coalitional support, networking opportunities, and other vital benefits (McCullough, 2008; McCullough et al., 2013). But the converse error poses no less a hazard: mistaking deliberate injury for mere accident is to court ruin or death. Individuals facing these crucial decisions are expected to integrate available informational cues relevant to the transgressor’s future intentions (Petersen et al., 2010; McCullough et al., 2013). Did the transgressor apologize for the act? Was there an expression of regret? Did the transgressor perhaps offer compensation or otherwise reliably signal an intention to avoid such harm in the future (Burnette et al., 2012; Tabak et al., 2012; Ohtsubo and Yagi, 2014). The victim’s empathy, sympathy, and perspective-taking abilities might also come into play: perhaps situational factors compelled the transgressor’s action, or the transgressor failed to realize the extent of the harm done to the victim (Petersen et al., 2010, 2012).

Processing these complex informational inputs requires representing the intentions, desires, emotions, and mental states of others, often termed a “theory-of-mind” or “mentalizing” ability. Theory of mind is the subject of considerable neuroscientific interest, and imaging studies repeatedly find co-activation of several brain regions during social cognition tasks associated with it (Lieberman, 2007; Van Overwalle, 2009). Central to the theory-of-mind network are the temporal-parietal junction (TPJ), medial prefrontal cortex (mPFC), precuneus, temporal poles, and superior temporal sulcus (STS), with the TPJ in particular appearing to play a major role in representing the belief states of others (Frith and Frith, 2003, 2006; Gallagher and Frith, 2003; Saxe and Kanwisher, 2003; Saxe et al., 2004; Perner et al., 2006; Lieberman, 2007; Aichhorn et al., 2009; Van Overwalle, 2009). Consistent with the evolutionary model of forgiveness, fMRI studies have sought and found evidence of heightened theory-of-mind activity during cognition related specifically to forgiving, much of it linked to the TPJ in particular. In their virtual ball-passing experiment, Will et al. (2014), for instance, operationalized forgiveness as participants acting equitably toward Cyberball players who had previously excluded them, vs. included them. fMRI scans showed that forgiveness was associated with increased activation of the bilateral TPJ and the dorsomedial prefrontal cortex (dmPFC)—regions that are regularly implicated in the mentalizing network, as the authors noted (Will et al., 2014).

In a series of neuroimaging studies of moral decision-making, Liane Young, Rebecca Saxe, and collaborators have marshaled evidence that the TPJ—specifically, the right TPJ (rTPJ)—plays a critical role in assessing the blameworthiness of actions performed by others, and that it does so by integrating information concerning the belief states of the actor. Across two studies (Young et al., 2007; Young and Saxe, 2009), fMRI scans recorded brain activity of subjects as they rated the blameworthiness of agents described in carefully matched narrative scenarios. The 2 × 2 design crossed the actions of the agents (harmful/not harmful) with the agent’s beliefs about the consequences of their actions (belief that the other person would be harmed/belief that the other person would not be harmed). As you’d expect, deliberately harmful actions were judged most blameworthy, while unintentional harm was deemed significantly less blameworthy, and TPJ activation was significant during every assessment of blameworthiness. Of particular interest, however, were findings that rTPJ activity was significantly elevated when the beliefs of the actor appeared to be in conflict with the outcomes of the action. That is, rTPJ activity was heightened when an actor harmed someone despite believing the action *would not* be harmful (Young and Saxe, 2009), or when an actor failed to harm someone, despite believing the action *would* be harmful. (Young et al., 2007). In the latter study, researchers determined that when subjects evaluated such accidental harms, the ultimate blameworthiness of the act exhibited a strong negative correlation with activation of the rTPJ. In other words, the less blameworthy the offense was judged to be, the greater the activation of the right TPJ. The authors accordingly suggested that the rTPJ indexes the extent to which individuals use information about the beliefs of others to find their harmful actions forgivable.

Studies using transcranial magnetic stimulation (TMS) to disrupt rTPJ functioning provide further evidence bolstering the view that the rTPJ influences moral judgment by integrating information about the harmfulness of action outcomes with information concerning actors’ belief states. In two additional experiments utilizing the 2 × 2 design outlined above, Young et al. (2010) demonstrated that disruption of rTPJ activity affected the degree to which participants incorporated information about the actor’s beliefs into judgment about the act’s blameworthiness. Specifically, when rTPJ function was disrupted vs. when it was not, participants tended to judge actions based solely on how harmful the outcome was, regardless of whether the actor envisioned and intended that outcome. With rTPJ activity impaired, participants deemed attempted (but failed) harms to be more permissible than otherwise.

Given the rTPJ’s involvement in assessing the blameworthiness of actions, might the region actually encode intentional vs. accidental harm in a spatially discernible pattern? Koster-Hale et al. (2013) explored this possibility in three experiments using multivoxel pattern analysis. The authors found that distinct spatial patterns of activity within the rTPJ were associated with narratives portraying intentional vs. accidental harms in neurotypical adults (as opposed to those with autism spectrum disorders). In addition, the individual differences in the magnitude of this neural pattern predicted individual differences in assigning blameworthiness: the stronger the pattern, the more forgiving the judgment of accidental harms. Finally, a separate experiment involving adults with autism spectrum disorder—a condition known to impair an array of theory-of-mind abilities (Baron-Cohen, 1997) including forgiveness for inadvertent harm (Moran et al., 2011)—found no evidence for encoding of intentional vs. accidental encoding of harms in the rTPJ or elsewhere, in line with behavioral data confirming that these autistic participants failed to modulate blame on the basis of actor intent. Converging lines of evidence thus support the notion that brain regions associated with theory of mind—and particularly the rTPJ—process the blameworthiness of others’ actions by taking into account both the harm that the act caused and the intentions that the perpetrator held. This body of evidence suggests that theory-of-mind functionality is associated with increased forgiveness for accidental harms, as well as with assigning blame to unsuccessful acts intended to cause harm.

Further evidence that forgiveness activates a theory-of-mind network comes from the work of Strang et al. (2014), whose results again suggest a crucial role for the TPJ. Strang et al. (2014) employed an ambiguous apology to examine the neural underpinnings of forgiveness. In their design, subjects (Player A) received payouts based on the answers other individuals (Player B) gave to trivia questions of moderate difficulty. If Player B answered correctly, Player A and Player B shared equal payouts of 100 points each, but if Player B answered incorrectly, Player A received only 50 points and was forced to make a decision regarding the payout made to Player B. Player A could “forgive” Player B, in which case Player B would receive the highest possible payout, 140 points; alternatively, if Player A chose not to forgive Player B, Player B received 110 points. Two items merit note. First, the payout structure incentivized Player B to answer incorrectly even if she knew the answer; second, Player B had the option of apologizing for missing the question and potentially influencing Player A’s forgiveness decision. The result of this design is a scenario in which Player A might suspect Player B of deliberately missing questions, costing Player A money to the benefit of Player B, and then offering an insincere apology. (Subsequent analyses suggested that Players B indeed did intentionally miss questions a significant portion of the time). fMRI results revealed that receiving an apology was accompanied by elevated activity of the left angular gyrus (a subsection of the TPJ), along with the left middle temporal gyrus and the inferior frontal gyrus. Forgiveness was associated with activation specifically of the right angular gyrus of the TPJ, which the authors found consistent with the notion of mentalizing taking place. The authors remarked, however, that they observed no activation of the mPFC and STS. Noting that these two regions are most often linked to sharing of emotions, the authors suggested that their design provided little or no information pertinent to the emotional state of the other players and hence elicited neural activity primarily related to cognitive rather than affective representations.

Final evidence concerning a crucial role of the TPJ, and perhaps especially the rTPJ, in theory-of-mind processes that impact forgiveness may come from neuroimaging investigations of a well-documented intergroup bias in costly third-party punishment, a bias known as “parochial punishment” (Baumgartner et al., 2012, 2013). Parochial punishment in these studies refers to the infliction of greater punishment upon outgroup members who have transgressed against ingroup members than upon ingroup members who have transgressed against outgroup members, even for the same harm (Baumgartner et al., 2012, 2013). Note that the punishment referred to here is administered by observers not directly involved in the transgression, rather than by victims directly harmed by the perpetrator (thus, it is “third-party” punishment rather than “second-party” punishment). Although third-party punishment is distinct from the purely dyadic interactions that form the basis of the evolutionary model of revenge and forgiveness highlighted in this paper, we suggest that the neural processes underlying it may well prove informative about mechanisms regulating dyadic revenge and forgiveness, for reasons elaborated below.

To explore the neural bases of parochial punishment, Baumgartner et al. (2012) utilized a third-party punishment task in which participants (whom we will call “punishers”) were given truthful information about the actions of fellow participants in a PD Game, including instances when fellow participants defected upon rather than cooperated with other players. Punishers were then provided a monetary endowment and offered a chance to spend some of that endowment to reduce the monetary rewards of one of the PD players (this was “punishment”). The researchers then systematically varied the ingroup/outgroup status of both punishers and the other participants. Behavioral results replicated numerous prior findings of the parochial punishment bias: punishment of outgroup members who defected on a cooperative ingroup member was substantially higher than punishment of ingroup members who defected on a cooperative outgroup member. Baumgartner et al. (2012) predicted—and found—that two distinct neural networks were active in conjunction with particular aspects of this punishment bias. First, increased punishment of outgroup members was associated with heightened activity of regions previously linked to two-party punishment— including the right dorsal caudate and right lateral prefrontal cortex, regions implicated in two-party punishment decisions as reviewed above. Second, and most crucial for present purposes, decreased punishment of ingroup members for the same offense was associated with elevated activity and connectivity of the bilateral TPJ and the dmPFC—the two nodes of the network associated with mentalizing. Baumgartner et al. (2012) argued that this pattern of findings was consistent with the view that mentalizing networks, involved in such activities as perspective-taking and understanding the intentions of others, might modulate the punishment of ingroup members.

Baumgartner et al. (2012)’s research indicated that neural networks associated with mentalizing underlie the third-party processes of parochial punishment, but these mechanisms might conceivably undergird revenge and forgiveness at the dyadic level as well. Specifically, if empathy, perspective-taking, and other mentalizing abilities associated with such regions as the TPJ and the dmPFC are associated with reduced punishment in the third-party scenarios examined by Baumgartner et al. (2012) they may also reduce punishment in two-way interactions by enabling victims to take the perspective of their transgressors, promoting better appreciation of the transgressor’s motives, intentions, and outcomes. Indeed, behavioral research on forgiveness has produced evidence that empathy toward the transgressor is an influential factor that promotes forgiveness in the wake of harm (McCullough et al., 1997, 1998, 2003; Worthington et al., 2000). Additional research supporting this hypothesis is Will et al.’s (2014) study of revenge and forgiveness following social exclusion, which found that self-reported perspective-taking correlated negatively with their measure of punishment. Taken together, these findings are consistent with the valuable relationships hypothesis advocated by the evolutionary model of forgiveness (McCullough, 2008; McCullough et al., 2013), given that fellow ingroup members are likely to be regarded in general as more valuable interaction partners than outgroup members. Indeed, the findings of Baumgartner et al. (2012) highlight a possible neural pathway by which more valued relationship partners—like the ingroup transgressors privileged in parochial altruism—could be more readily forgiven. On this account, theory-of-mind processes, including those in the TPJ, lead to greater forgiveness in valued relationships by promoting greater perspective-taking toward those partners relative to less valued individuals—essentially generating an empathy bias which would in turn down-regulate punitive motivations toward close associates.

The picture is somewhat muddied, however, by a subsequent study conducted by Baumgartner et al. (2013), who used TMS to test the hypothesis that theory-of-mind processing specifically in the TPJ plays a causal role in parochial punishment. Baumgartner et al. (2013) employed essentially the same third-party punishment paradigm as the 2012 study, but randomly assigned punishers to one of three groups: those with rTPJ function disrupted by TMS; those with left TPJ function disrupted by TMS; and those in a sham TMS condition. Baumgartner et al. (2013) again replicated behavioral findings of a parochial punishment bias. More crucially, TMS results suggested that the parochial punishment bias was moderated by rTPJ activity: the treatment group with disrupted rTPJ activity showed significantly less parochial punishment bias, relative to treatment groups in which the left TPJ was disrupted, or in which sham TMS took place. Moreover, differences in self-reported retaliatory motivation mediated the differences in parochial punishment bias. As a final twist, the researchers captured self-report data on the extent to which participants felt able to take the perspective of both ingroup and outgroup transgressors. Participants with disrupted rTPJ function reported no differences in ability to take the perspective of an ingroup vs. an outgroup transgressor; individuals with sham disruption, or disruption of the left TPJ, by contrast reported that they could more readily relate to transgressors who were ingroup rather than outgroup members. Follow-up analyses revealed that perspective-taking differences mediated the effect of rTPJ disruption on retaliatory motivation, which in turn mediated the effect on parochial punishment.

To this point, the findings are consistent with the hypothesis that perspective-taking abilities associated with the rTPJ promote greater empathy toward ingroup members, down-regulating punitive motivation toward ingroup members who harm outgroup members, relative to outgroup members who harm ingroup members. Yet it is also possible that rTPJ activity is associated instead with upregulating punitive motivation toward outgroup members, rather than downregulating it toward the ingroup. Follow-up analyses of self-report data tended to support this latter alternative: participants with disrupted rTPJ function reported reduced motivation to retaliate against outgroup members, relative to participants with disrupted lTPJ function or those in the sham treatment group; there was no evidence of increased punitive motivation toward ingroup members in conjunction with impaired rTPJ function. If these findings prove robust, they may suggest a complex role for empathy and perspective-taking when intergroup relationships are highly salient. In such contexts, perspective-taking biased toward ingroup members might well increase outgroup-directed revenge rather than increase ingroup-directed forgiveness. We note again that the studies conducted by Baumgartner et al. (2012, 2013) involve third-party rather than dyadic interactions. Future TMS research involving specifically one-on-one interactions could clarify whether the TPJ or perhaps other regions in the mentalizing network play a causal role in generating transgressor-directed empathy in such dyadic interactions, whether empathy is more easily directed toward valuable relationship partners, and whether such empathy is in turn associated with increased forgiveness, consistent with prior behavioral studies.

In this discussion of theory-of-mind processing, we have highlighted the role of the TPJ, and particularly the rTPJ. We conclude this section by noting two studies implicating other theory-of-mind regions in forgiveness, specifically the mPFC and precuneus. Yamada et al. (2012) investigated forgiveness in the context of criminal law, asking mock jurors to consider reducing the sentences of hypothetical defendants judged guilty of murder, using scenarios designed to arouse or not arouse the subject’s sympathy for the defendant. fMRI results associated sympathy with more intense activity in the precuneus, dmPFC, and left TPJ, while the dmPFC, precuneus, and TPJ were also associated with granting reduced sentences (as was the dACC). Activation of this broad theory-of-mind network during decisions to mitigate criminal sentences is consistent with the view that forgiveness recruits a wide array of mentalizing abilities, including perspective-taking (associated particularly with the precuneus), empathy (associated particularly with the dmPFC), and inferring the intentions of others (associated particularly with the TPJ) (Yamada et al., 2012). Further neuroscientific evidence suggesting a role of mentalizing in forgiveness comes from Ricciardi et al. (2013). In a study of forgiveness using imagined social scenarios, Ricciardi et al. (2013) reported activation of the precuneus in contexts where participants had to interpret the motivations of their imagined transgressor, leading the authors to interpret this activity as a sign of perspective-taking, consistent with prior work on the precuneus (Cavanna and Trimble, 2006).

## Discussion and Future Research

The neuroscientific research reviewed here sheds light on the neural systems that may instantiate the adaptive information-processing mechanisms hypothesized by evolution-minded researchers to underlie forgiveness and revenge. Such research provides evidence linking vengeful motivation to a reward-based brain network, identifies prefrontal areas consistent with inhibition of retaliatory sentiment, and correlates the activity of a theory-of-mind network with assessments of transgressor intentions and blameworthiness. Given that forgiveness remains a fairly understudied topic within psychology (McCullough et al., 2013), and that (perhaps as a result) neuroscientific studies dedicated to forgiveness are relatively few, the progress reflected in this literature is encouraging. Nonetheless, both the neuroscience and the evolutionary psychology of forgiveness are fledgling fields, simultaneously confronting an abundance of opportunity and an array of obstacles. The cognitive neuroscience of forgiveness, as its practitioners have noted, faces a number of limitations. For one thing, the cognitive neuroscientific studies conducted thus far examine forgiveness using diverse experimental methods that often render comparisons of results problematic (Strang et al., 2014). In addition, these studies suffer from small sample sizes (Brüne et al., 2013; Ricciardi et al., 2013), and often lack sufficient ecological validity (Strang et al., 2014).

To these concerns, we would add the following. From virtually the beginning of scientific interest in the topic, forgiveness has been conceptualized as *change* in interpersonal motivation, and thus as a construct which benefits from being modeled and analyzed in explicitly temporal terms (McCullough et al., 2000, 2003). Behavioral research on forgiveness (e.g., McCullough et al., 2003), including several longitudinal studies undertaken to test the evolutionary model highlighted here (McCullough et al., 2010, 2014), has embraced this longitudinal design challenge. In this work, researchers have introduced latent growth models that operationalize forgiveness as linear or logarithmic change (depending on the time scale) and capture decline over time in punitive and avoidant sentiment toward the perpetrator following a transgression. Such models distinguish the participant’s declining motivational curve (“trend forgiveness)” from low levels of punitive or avoidant sentiment that might immediately follow the transgression (“forbearance”) (McCullough et al., 2003). To the best of our knowledge, no neuroscientific study has yet examined the neural correlates of forgiveness operationalized as latent change in interpersonal motivation over time. Indeed, neuroscientific studies that address revenge and forgiveness are decidedly cross-sectional, capturing brief neural states shortly after a transgression [for one exception involving forgiveness judgments in individuals with PTSD, see Farrow et al. (2005)]. In this sense, the bulk of the neuroscientific literature may be studying forbearance (a key component of forgiveness, to be sure) but illuminating to a much lesser extent forgiveness understood as change over time. The existing neuroscientific studies of forgiveness have rarely if ever determined whether their neural snapshots predict motivational change in participants over subsequent days, weeks, or months. Lacking, too, are imaging studies that attempt to identify changes in brain activity over time in select regions that might correlate with changes over time in observed transgression-related motivation (an undoubtedly daunting task).

As a final set of concerns, we note that most existing neuroscientific studies—e.g., the UG studies (Sanfey et al., 2003; Tabibnia et al., 2008; Brüne et al., 2013), the Cyberball study of Will et al. (2014), and even the trivia question paradigm of Strang et al. (2014)—examine a limited set of generally minor transgressions. Indeed, the vast majority of neuroscientific studies of revenge and forgiveness employ a behavioral economics framework, within which transgressions are characterized at best by the loss of a few dollars that participants didn’t possess prior to the experiment. Often missing from these studies are the types of transgressions reported in longitudinal research into forgiveness (e.g., McCullough et al., 2003, 2014; Tabak et al., 2012): romantic infidelity, betrayals of confidence, insults, rape, and neglect. Longitudinal studies of the transgressions that arise in real life diverge from most manufactured laboratory contexts in at least two crucial ways. First, real-life transgressions are in most cases likely to be more severe than the minimal monetary losses incurred in behavioral economics experiments. The average transgression severity reported in Tabak et al.’s (2012) longitudinal study, for instance, was 4.84 out of a possible 6, with 6 designating “the worst pain I ever felt.” A few defections in a PD Game are unlikely to evoke anything like the level of hurt experienced by a betrayed spouse or neglected friend. Second, as the example of the betrayed spouse also reminds us, the real-life transgressions that require forgiveness typically do not involve anonymous strangers in one-shot interactions. Instead, they often involve the people closest to us—our friends, our family, our romantic partners. These disparities between real-life transgressions and the behavioral economics tasks that predominate in neuroscientific studies of revenge and forgiveness raise the possibility that we may be failing to observe the brain activity accompanying forgiveness as it most often occurs in the real world (though this possibility is of course not unique to forgiveness research).

These gaps in the existing neuroscience of forgiveness provide clear opportunities for future research. Among these are, first, investigating neural activity associated with transgressions involving relatives, romantic partners, and close friends, in addition to anonymous strangers. Second, studies should attempt to explore transgressions more serious than what takes place during behavioral economics tasks such as the PD or UG. Although more serious harms obviously cannot be experimentally manufactured, in principle it may be possible to expand transgression-recall techniques, such as those employed by McCullough et al. (2003, 2010, 2014), into a neuroscientific context. Neuroscientific studies involving recalled interactions with recent transgressors offer the prospect of uncovering additional neural mechanisms of forgiveness as yet unilluminated by a literature largely reliant upon behavioral economics. Third, longitudinal methods could remedy the current lack of studies attempting to identify the neural correlates of forgiveness operationalized as change taking place over significant periods of time—days, weeks, and months. Fourth, existing neuroscientific studies have utilized a wide array of tools to explore moral judgment, blame, and other issues relevant to forgiveness, as our discussion of theory-of-mind research hopefully made clear. Nevertheless, the diversity of neuroscientific methods—including lesion studies, disruption by TMS, and the study of high-functioning autistic individuals—has perhaps not been fully brought to bear in studies where actual change in interpersonal motivation over time is the key outcome measure. One worthwhile possibility might be to build on Baumgartner et al.’s (2013) study of parochial altruism by using TMS to determine if TPJ activity promotes forgiveness of high-value vs. low-value relationship partners via increased perspective-taking.

Beyond addressing the challenges noted above, several other avenues toward progress present themselves. This review of the literature suggests that no neuroscientific imaging study of forgiveness has used the instruments developed and employed by researchers investigating the topic from an evolutionary perspective. Of particular note in this context is the Transgression Related Interpersonal Motivations (TRIM) scale (McCullough et al., 2006), which uses three subscales (revenge, avoidance, and benevolence) to assess the distinct motivational components hypothesized to form the basis of forgiveness. Use of the TRIM scale in future neuroscientific studies of forgiveness would provide a much-needed supplement to a literature that operationalizes forgiveness primarily in behavioral terms, most often as monetary decisions in behavioral economics games. For example, in their Cyberball study, Will et al. (2014), defined forgiveness as a fair allocation of money to transgressor during a DG, while acknowledging that additional research was needed in order to demonstrate the measure’s relationship to motivation. Incorporating more fine-tuned measures of subjective interpersonal motivations into their studies would enable neuroscientists to provide a richer description of the qualitative states accompanying the neural systems associated with behavioral forgiveness, and secondly to verify more thoroughly that behavioral forgiveness is indeed linked with the suite of shifting interpersonal motivations predicted by theory. Finally, more widespread use of such measures might facilitate research of scenarios in which subjective motivations and behavior may be in conflict—for instance, when the relatively greater power of a transgressor may compel cooperative behavior, despite the victim’s strong motivation to retaliate.

With its three subscales, the TRIM scale also provides an opportunity to address the existing literature’s perhaps excessive focus on punishment in response to harm. Few if any neuroscientific studies examine avoidant motivations following a transgression; indeed, most experimental paradigms require victims to interact with the transgressor in some manner (e.g., punish or treat fairly), without allowing for motivations to withdraw or engage with other, preferred partners. Use of the TRIM scale might thus illuminate the brain systems associated with avoidant as well as punitive motivations in conjunction with decision-making following interpersonal harm.

Another instrument developed by evolutionary researchers is the RVEX scale (Burnette et al., 2012), used to measure the hypothesized predictor variables relationship value and exploitation risk. Evolutionary neuroscientists might employ the RVEX scale in future imaging studies, with the expectation that varying scores on the instrument’s two subscales might correspond to activation of two distinct functional networks. High scores on the exploitation subscale of the RVEX measure (indicating high expected probability of future costs imposed by the transgressor) might be associated with increased activation of the amygdala, known to be deeply engaged in issues of trust (Rilling and Sanfey, 2011) and fair treatment (Lieberman, 2007). Likewise, high scores on the relationship value subscale might be associated with increased activation of the vmPFC, which has been linked to high valuation of long-term benefits, particularly those derived from cooperative interaction (Rilling and Sanfey, 2011).

Neuroimaging techniques employing the measures discussed above might also be integrated with a robust literature examining the neurochemical basis of social-decision making. For instance, prior research suggests that oxytocin may down-regulate fear responses in the amygdala, including responses specifically tied to social betrayal (Rilling and Sanfey, 2011). If so, exogenous oxytocin administered to subjects during neuroimaging studies of forgiveness might be associated with decreased activation of the amygdala, lower scores on the RVEX exploitation scale, and higher levels of forgiveness on the TRIM scale, relative to subjects administered a placebo. Such a result would suggest that amygdala activity, as regulated by oxytocin, mediates the effect of exploitation risk on forgiveness. Research also suggests a role for serotonin in the vmPFC, a region linked to valuing long-term benefits derived from cooperation (Rilling and Sanfey, 2011). If serotonin plays an important role on the valuation of long-term cooperative benefits, variation in serotonin levels during neuroimaging studies of forgiveness might be associated with increased activation of the vmPFC, high scores on the relationship value subscale of the RVEX measure, and higher levels of overall forgiveness on the TRIM scale.

Finally, behavioral research on forgiveness has long prioritized the perspective of the victim, while largely neglecting to consider transgressors seeking forgiveness from those whom they have harmed (McCullough et al., 2000). A recent study examining the use of costly apologies by offenders (Ohtsubo and Yagi, 2014) may or may not signal a shift of emphasis toward transgressors in the behavioral literature, but the neuroscientific literature has evinced little interest in the viewpoint of the offender thus far. If cognitive mechanisms for forgiveness benefit victims who successfully restore damaged relationships, similar mechanisms should regulate the actions of transgressors who face punishment or avoidance from valuable others. Decisions to seek forgiveness should be adaptively regulated just as much as decisions to grant it, and neuroscience may yet illuminate the brain systems that underlie these fundamental choices.

## Summary

We have presented in brief the dominant evolutionary model of forgiveness, and have reviewed the neuroscientific literature on revenge and forgiveness, with the goal of using each body of work to interrogate and illuminate the other. The neuroscientific findings presented here identify neural systems that may reflect the computational systems posited by the evolutionary model. A broad body of neuroscientific research links retaliatory motivation to reward-based areas of the brain, singles out prefrontal areas likely associated with inhibition of retaliatory sentiment, and correlates the activity of a theory-of-mind network with assessments of the intentions and blameworthiness of harmdoers. In addition, we have sought to identify gaps in the existing literature, and have proposed future research directions that might address them, at least in part. We suggest in particular the value of using the RVEX and TRIM measures in future neuroscientific work, and the need to incorporate longitudinal methods that may allow researchers to identify the neural correlates of forgiveness operationalized as change in interpersonal motivation over time.

## Author Contributions

JB conceived, designed, and drafted the review; gives final approval of the version to published; and agrees to be held accountable for all aspects of the work. EL contributed substantially to the work; revised it critically for important content; gives final approval of the version to be published; and agrees to be held accountable for all aspects of the work.

## Conflict of Interest Statement

The authors declare that the research was conducted in the absence of any commercial or financial relationships that could be construed as a potential conflict of interest.
